# The CORONIS Trial. International study of caesarean section surgical techniques: a randomised fractional, factorial trial

**DOI:** 10.1186/1471-2393-7-24

**Published:** 2007-10-22

**Authors:** The CORONIS Trial Collaborative Group

**Affiliations:** 1 National Perinatal Epidemiology Unit, University of Oxford, Old Road Campus, Oxford, OX3 7LF, UK

## Abstract

**Background:**

Caesarean section is one of the most commonly performed operations on women throughout the world. Rates have increased in recent years – about 20–25% in many developed countries. Rates in other parts of the world vary widely.

A variety of surgical techniques for all elements of the caesarean section operation are in use. Many have not yet been rigorously evaluated in randomised controlled trials, and it is not known whether any are associated with better outcomes for women and babies. Because huge numbers of women undergo caesarean section, even small differences in post-operative morbidity rates between techniques could translate into improved health for substantial numbers of women, and significant cost savings.

**Design:**

CORONIS is a multicentre, fractional, factorial randomised controlled trial and will be conducted in centres in Argentina, Ghana, India, Kenya, Pakistan and Sudan.

Women are eligible if they are undergoing their first or second caesarean section through a transverse abdominal incision.

Five comparisons will be carried out in one trial, using a 2 × 2 × 2 × 2 × 2 fractional factorial design. This design has rarely been used, but is appropriate for the evaluation of several procedures which will be used together in clinical practice. The interventions are:

• Blunt versus sharp abdominal entry

• Exteriorisation of the uterus for repair versus intra-abdominal repair

• Single versus double layer closure of the uterus

• Closure versus non-closure of the peritoneum (pelvic and parietal)

• Chromic catgut versus Polyglactin-910 for uterine repair

The primary outcome is death or maternal infectious morbidity (one or more of the following: antibiotic use for maternal febrile morbidity during postnatal hospital stay, antibiotic use for endometritis, wound infection or peritonitis) or further operative procedures; or blood transfusion.

The sample size required is 15,000 women in total; at least 7,586 women in each comparison.

**Discussion:**

Improvements in health from optimising caesarean section techniques are likely to be more significant in developing countries, because the rates of postoperative morbidity in these countries tend to be higher. More women could therefore benefit from improvements in techniques.

**Trial registration:**

The CORONIS Trial is registered in the Current Controlled Trials registry. ISCRTN31089967.

## Background

Caesarean section is one of the most commonly performed abdominal operations on women in most countries of the world. Rates have increased markedly in recent years, and are about 20–25% in many developed countries [1,2]. The rates in other parts of the world vary widely, from 1.6% in Haiti to 59% in Chilean private hospitals [3].

According to a population-based cross sectional study in Madras, India [4], caesarean section rates in the public, charitable and private sectors were 20%, 38% and 47% respectively, and the total population caesarean section rate was 32.6% with a primary caesarean section rate of 25%. With a private sector that accounts for 50–70% of patient care currently, and public sector health financing decreasing, the private sector is likely to dominate health care in India in the future, which means that the general rate of caesarean section will rise substantially. This rising trend is being observed in many other countries.

A variety of surgical techniques for all elements of the caesarean section operation are in use [5]. Many of these have not yet been rigorously evaluated in randomised controlled trials, and it is not known whether any are associated with better outcomes for the women and babies. Because of the huge number of women that undergo caesarean section, even small differences in post-operative morbidity rates between techniques could translate into improved health for a substantial number of women, and significant savings of costs and resources for health services. It is therefore important that caesarean section operations are performed as safely and effectively as possible and that methodologically rigorous randomised controlled trials are performed to establish the effectiveness of different surgical techniques.

Improvements in health from optimising caesarean section techniques are likely to be more significant in developing countries, because the rates of postoperative morbidity in these countries tend to be higher. More women could therefore benefit from improvements in techniques.

The aim of this study is to examine five specific aspects of caesarean section technique to help determine which methods lead to an optimum outcome for women and their babies.

These interventions are:

1. Blunt versus sharp abdominal entry

2. Exteriorisation of the uterus for repair versus intra-abdominal repair

3. Single versus double layer closure of the uterus

4. Closure versus non-closure of the peritoneum (pelvic and parietal)

5. Chromic catgut versus Polyglactin-910 for uterine repair

## Techniques for caesarean section

### Blunt versus sharp abdominal entry (Table 1)

**Table 1:** Blunt versus sharp abdominal entry

**Study**	**n**	**Infection**		**Pain**		**Health service use**		**Other outcomes**	
		Outcome	Result	Outcome	Result	Outcome	Result	Outcome	Result

**Decavalas**^7^	268				Operating time (mean)	26 v 35 min	Composite morbidity (RR)	0.51 (0.24, 1.05)	
**Mathai et al**^8^	101	Febrile morbidity (RR)	0.25 (0.07, 0.82)	Analgesia in first 4 hours post op (RR)	0.55 (0.40, 0.76)	Operating time (mean (SD))	33 (7.8) v 44 (16.9) min		
**Franchi et al**^9^	310					Operating time (median (range))	30 (12–60) v 32 (18–70) min	Composite morbidity (RR)	0.94 (0.39, 2.24)

A protocol for a systematic review of abdominal incisions for caesarean section has been published in the Cochrane Library, and the full review is in progress [6].

Three randomised controlled trials have been published that compared the 'blunt' (sometimes referred to as the Joel-Cohen) method of abdominal entry with the 'sharp' (sometimes referred to as the Pfannenstiel) incision. The study by Decavalas et al [7] which included 268 women was only reported in abstract form and therefore few details of the methodology were available. The outcomes reported suggested a reduction in operating time, an improvement in a composite measure of maternal morbidity (unspecified) and fewer adhesions when a repeat operation was performed, with the Joel-Cohen method. The study by Mathai et al [8] randomised 101 women and found a marked improvement in the need for analgesia in the first four postoperative hours, a shorter operating time and lower febrile morbidity with the Joel-Cohen technique. This study used a standard spinal anaesthetic for all women to make comparisons of analgesic requirements easier. However, the third study, by Franchi et al [9], which included 310 women, found no difference between the Joel-Cohen and Pfannenstiel groups.

Overall, these studies, which recruited a total of 679 women, may suggest some benefits of the Joel-Cohen approach, but they have not yet been formally synthesised in a systematic review, and there appear to be unexplained inconsistencies between the results of the three studies.

A number of randomised trials have evaluated combinations of techniques, such as the Misgav-Ladach method for caesarean section and are the subject of a Cochrane systematic review which is in protocol stage [10]. These frequently compare a method involving blunt abdominal entry with one using sharp abdominal entry. However, the techniques being compared also differ in other respects (such as single or double layer uterine closure and whether or not the peritoneum is closed) and therefore it is not possible to separate the effects of blunt or sharp abdominal entry from those of the other techniques. These trials therefore do not provide any information relevant to this comparison.

### Exteriorisation of the uterus for repair versus intra-abdominal repair (Table 2)

**Table 2:** Exteriorisation of the uterus for repair versus intra-abdominal repair

**Study**	**n**	**Infection**		**Wound complications**		**Blood loss**		**Health service use**	
		Outcome	Result	Outcome	Result	Outcome	Result	Outcome	Result

**Cochrane review**^11^	1,294	Fever (>3 days) (n = 308) (RR)	0.41 (0.17, 0.97)	Infection, haematoma, breakdown (n = 735) (RR)	0.88 (0.53, 1.46)	Vol blood lost (n = 504) (WMD)	17.11 (-23.15, 57.37) ml	Hospital stay (n = 766) (WMD)	0.24 (0.08, 0.39) days
		Endometritis (n = 592) (RR)	1.29 (0.64, 2.60)			Drop in haematocrit (n = 324) (WMD)	-0.47 (-1.48, 0.54)%		

The Cochrane systematic review comparing exteriorisation with intraperitoneal repair found six trials with 1294 women in total [11].

This review suggested a reduction in early febrile morbidity in the exteriorisation group. Although this finding was statistically significant (RR 0.41, 95% CI 0.17–0.97), these results were based on only one trial of 308 women, which was of poor methodological quality. The length of hospital stay was found to be marginally longer in the exteriorisation group in the four trials assessing this outcome (WMD 0.24 days, 95% CI 0.08–0.39), which may not have much clinical significance. No difference in the incidence of endometritis or wound infection was found between the two groups.

Pain was addressed by only one study [12], and this used 6 different scoring systems to do so. One of these showed a statistically significantly difference between groups, but a more reliable measurement may have been a statistical test incorporating all six readings into one test.

Overall the evidence for the harms or benefits of exteriorisation of the uterus for repair is poor due to the small size of the studies and the diversity of the outcomes examined.

### Closure versus non-closure of the peritoneum (pelvic and parietal) (Table 3)

**Table 3:** Closure versus non-closure of the peritoneum

**Study**	**n**	**Infection**		**Blood loss**		**Health service use**		**Other outcomes**	
		Outcome	Result	Outcome	Result	Outcome	Result	Outcome	Result

**Cochrane review**^13^	1,811	Postoperative fever (n = 874) (RR)	0.66 (0.46, 0.95)			Operating Time (n = 974) (WMD)	-7.33 (-8.43, -6.24) min	Analgesic dose required (n = 393) (WMD)	-0.29 (-0.69, 0.12)
		Endometritis (n = 474) (RR)	1.30 (0.62, 2.71)			Postoperative days in Hospital (n = 974) (WMD)	-0.39 (-0.51, -0.28)		
		Wound infection (n = 534) (RR)	0.60 (0.29,1.21)						

The Cochrane review of closure versus non-closure of the peritoneum, visceral and/or parietal peritoneum was last updated in 2003. It incorporates nine trials including 1,811 women comparing non-closure versus closure of the visceral and/or the parietal peritoneum [13]. This review found that non-closure was associated with a reduction in operating time (WMD -7.33 mins, 95% CI -8.43, -6.24) and postoperative fever (RR 0.66, 95% CI 0.46, 0.95), but no significant difference in the number of analgesic doses (WMD -0.29, 95% CI -0.69, 0.12), wound infection (RR 0.60, 95% CI 0.29, 1.21) or endometritis (RR 1.30, 95% CI 0.62, 2.71). However, the trials were generally small and of variable methodological quality; three of them used quasi-randomisation methods and hence did not have secure concealment of allocation, rendering them at high risk of bias.

A long-term follow-up study of one of the trials included in the Cochrane review has also been published [14]. 144 out of 280 women originally randomised were included in this study (51%). There were no differences in fertility, abdominal pain, urinary symptoms, or adhesions at subsequent surgery, but the small sample size would be very unlikely to detect any but the largest differences.

These studies suggest that non-closure of the peritoneum may carry some short-term advantages, including a lower risk of post-operative infection, shorter operating time and shorter hospital stay. Again, however, the studies identified were small and the methodology was not always strong.

### Single versus double layer closure of the uterus (Table 4)

**Table 4:** Single versus double layer closure of the uterus

**Study**	**n**	**Infection**		**Blood loss**		**Health service use**		**Other outcomes**	
		Outcome	Result	Outcome	Result	Outcome	Result	Outcome	Result

**Cochrane review**^15^	1,006	Endometritis (n = 784) (RR)	1.22 (0.91–1.63)	Transfusion (n = 906) (RR)	0.80 (0.34–1.92)	Operating time (Mean)	43.8 v 47.5 min	Moderate or major deformity of	0.19 (0.06–0.60)
				Extra haemostatic sutures (n = 906) (RR)	0.93 (0.79–1.08)			scar on radiography (n = 100) (RR)	
				>8% drop in haematocrit (n = 906) (RR)	1.09 (0.86–1.38)				
**Heidenreich**^17^	125	Post-operative fever	4.8% v. 7.2%	Transfusion	3.8% v. 3.2%			Scar thickness by US (8–10 days after surgery)	0.72 v. 0.82 cm
**Sood**^18^	208	Febrile morbidity (RR)	0.50 (0.27–0.94)	Intraoperative blood loss (Mean)	600 v. 629.6 ml	Operating time (Mean) Hospital stay (Mean)	31.3 v. 33.1 min 6.67 v. 7.19 days	Pain (VAS Score) (Mean)	2.91 v. 3.05
		Endometritis (RR)	0.44 (0.20–0.95)	Additional suture (RR)	1.36 (0.85–2.17)				
		Wound infection (RR)	0.46 (0.15–1.45)						

The Cochrane systematic review of single versus double layer closure of the uterine incision includes two trials which involve 1006 women in total, although each outcome has data from only one trial [15]. This review has not been updated since 1996, but a protocol for an updated and revised review has been published and the review is in progress [16]. Two further trials comparing single and double layer uterine closure published since the Cochrane review was last updated was identified, involving 333 women [17,18]. None of the existing studies suggests major differences in outcomes between single and double layer uterine closure.

Chapman et al [19] measured maternal outcomes in the subsequent pregnancy of women included in one of the studies in the Cochrane review. There was no detectable difference in the length of labour, mode of delivery, risk of scar dehiscence or haemorrhage between the two groups. However, the numbers were small and only a small proportion of the women randomised could be included in this follow-up study (145 women followed up out of a total study population of 906 women).

Two observational studies of the long-term effects of single and double layer uterine closure have been published [20,21]. Bujold et al examined a retrospective cohort of 2,142 women from one hospital in Canada who had undergone a trial of labour following a single previous lower segment caesarean section between 1988 and 2000. The main finding was an increased risk of uterine rupture with previous single layer closure. However, other differences may account for the differences in outcome in this observational study. For example, the caesarean technique used changed from mainly double layer closure to mainly single layer closure during the period studied, and the selection criteria for trial of labour may have changed in this time too. Thus women who had single layer closure may have been more likely to have a trial of labour. This study's result was not replicated by a second observational study, which found no increased risk of uterine rupture with single layer closure [21].

Overall there is little evidence that there is any difference in short term outcomes between single and double layer uterine closure. The observational study by Bujold et al has raised the possibility of an increased risk of uterine rupture in labour after single layer closure, and further research using larger, randomised controlled studies is needed to investigate this possible association.

### Chromic catgut versus Polyglactin-910 for uterine repair

Materials for suturing the uterus are included in a recently published Cochrane review [22]. This review was not able to identify any published or ongoing trials comparing different suture materials for this element of the caesarean section operation.

## Summary

The existing trials provide only limited evidence about the advantages and disadvantages for mother and baby of the different options for the conduct of caesarean sections.

All have sample sizes that are insufficient to detect clinically or economically important differences between the groups. Given the very high number of caesarean sections performed, even small differences may be important for the cost of health services or the population's health. Reliable detection of differences of this size is likely to require a sample size of several thousand, but the largest published trial has a sample size of 906 [23].

Many of the trials measure surrogate outcomes such as duration of operation which may have no correlation with maternal or neonatal outcome. Substantive measures of morbidity such as antibiotic use, fever, post-operative pain and the wellbeing of the infant are more likely to provide guidance for clinical decisions in the future, which is why the CORONIS Trial has chosen to measure these outcomes.

The methodology of the existing trials is generally poor or inadequately described. For example, quasi-randomisation by hospital number or date, non-blinded assessment of outcomes and exclusion of randomised women from the analysis are found in some of these studies, and all of these practices could lead to bias. The results reported by many of the existing studies may therefore be unreliable. This may be exacerbated by combining such unreliable studies in meta-analyses, which may in turn give a more precise but still biased summary.

## Objectives

The objectives of the trial are to determine whether there are any differences in maternal morbidity when comparing the following five pairs of alternative surgical techniques undertaken during the time of caesarean section:

1. Blunt versus sharp abdominal entry

2. Exteriorisation of the uterus for repair versus intra-abdominal repair

3. Single versus double layer closure of the uterus

4. Closure versus non-closure of the peritoneum (pelvic and parietal)

5. Chromic catgut versus Polyglactin-910 for uterine repair

## Study design

The study is a multicentre, fractional factorial randomised controlled trial and will be conducted in centres in the following six countries: Argentina, Ghana, India, Kenya, Pakistan and Sudan. The collaborating institutions are centres with experience in conducting trials. These centres also have experience in detailed follow-up of large numbers of women.

The study will not alter or interfere with any treatment or care given routinely to women in the hospitals where the caesarean section takes place except on the interventions to be evaluated.

### Factorial design

Factorial trials maximise the efficiency of a trial by including more than one trial question into a single trial population. Instead of having one trial which compares, for example, single versus double layer closure of the uterine incision and another trial comparing closure versus non-closure of the peritoneum, both comparisons can be combined into one trial using only the number of women necessary to answer one of these questions in isolation.

In the CORONIS Trial five comparisons will be carried out in one trial, using a 2 × 2 × 2 × 2 × 2 factorial design. Such a design has rarely been used [24,25], but is appropriate for the evaluation of several procedures which will be used together in clinical practice. In this trial of different caesarean section techniques, using five pairs of possible allocated interventions (1 versus "not 1", 2 versus "not 2", 3 vs versus "not 3", 4 versus "not 4", 5 versus "not 5"), participants can receive one of 32 possible alternatives.

In the analysis of a factorial trial the same process is used for a 2 × 2 × 2 × 2 × 2 factorial design as for a 2 × 2 factorial design. All those allocated 1 are compared with all those allocated "not 1" regardless of what other interventions were allocated, provided that there is no interaction. This is best illustrated using an example. Consider a 2 × 2 factorial trial comparing the effects of single versus double layer closure of the uterine incision and closure versus non-closure of the peritoneum. Hence, there are four groups in the trial:

• single layer and closure of the peritoneum

• single layer and non-closure of the peritoneum

• double layer and closure of the peritoneum

• double layer and non-closure of the peritoneum

A simple example (Table 5) of no interaction being present. Here, the febrile morbidity risk ratio for double versus single layer uterine closure is the same in those who had closure of the peritoneum (20% vs 10% gives risk ratio = 2) and those who had non-closure (30% vs 15% gives risk ratio = 2). Therefore, the single layer and double layer groups may be combined and the analysis is based on the (bold) marginal totals (25% vs 12.5% gives risk ratio = 2).

**Table 5:** Example of no interaction present

	**Single**	**Double**	**Total**
**Closure**	10/100 (10%)	20/100 (20%)	**30/200 (15%)**
**Non-closure**	15/100 (15%)	30/100 (30%)	**45/200 (22.5%)**
**Total**	**25/200 (12.5%)**	**50/200 (25%)**	**75/400 (18.75%)**

Numbers shown are number of cases of febrile morbidity/total (percentage)

Similarly, Table 6 gives a simple example of an interaction being present. Here, the febrile morbidity risk ratio for double versus single layer uterine closure is much smaller in those who had closure of the peritoneum (10% vs 10% gives risk ratio = 1) than in those who had non-closure (40% vs 20% gives risk ratio = 2). Therefore, we would present these two risk ratios separately rather than using the combined data.

**Table 6:** Example of interaction present

	**Single**	**Double**	**Total**
**Closure**	**10/100 (10%)**	**10/100 (10%)**	20/200 (10%)
**Non-closure**	**20/100 (20%)**	**40/100 (40%)**	60/200 (30%)
**Total**	30/200 (15%)	50/200 (25%)	50/400 (12.5%)

Numbers shown are number of cases of febrile morbidity/total (percentage)

### Fractional Factorial Trials

In a standard 2 × 2 × 2 × 2 × 2 factorial design, all participants would receive one of 32 possible alternatives, and each of these alternatives would include five interventions. This study is a fractional factorial design, and, therefore, each participant will be allocated only three out of the five possible interventions. Such an incomplete factorial design was considered appropriate because it allows five surgical techniques to be tested in the same trial, but restricts the number of techniques being tested per centre and per surgeon to three. Hence, each centre needs to implement training for three (not five) surgical techniques and each surgeon needs to remember the three (not five) allocated techniques for every randomised woman. There are two main implications of this incomplete factorial design. First, in the study design, we will ensure that a similar number of women are included in each of the five comparisons (see later section on Feasibility). Second, in the analysis, we will not be able to test for interactions between more than three interventions; this won't pose a problem because we are only planning to test for pairwise interactions (see later section on Analysis).

## Treatment allocation

Randomised allocation will be achieved by a variety of methods. For centres with reliable telephone access, there will be central telephone randomisation, using a web-based system and a toll-free telephone number. This will be a 24/7 system which will be attended by the local Regional Trial Office Co-ordinator during day time hours and will use a voice recognition system over night. The system will allocate a number which will correspond to a series of allocation envelopes held at the participating site.

Each envelope will contain the allocation for a woman which will be recorded on the data collection form and the envelope kept for verification.

In centres with unreliable telephone access, the only available option is to use a sequentially numbered series of sealed opaque envelopes.

The allocation will be to three options. This will be to one or other of three of the interventions. For example, the allocation may be to:

"Sharp abdominal entry; single layer repair of uterus; non-closure of the peritoneum."

All randomisation data will be recorded and held centrally at the International Co-ordinating Centre in NPEU.

### Minimisation

As the sample size of the trial is large, chance imbalances between groups for major prognostic variables are unlikely. However, minimisation will be used to ensure balance between groups with respect to the most important prognostic risk factor: in-labour and not in-labour caesarean section. In addition, for centres using telephone randomisation, minimisation will be used to ensure balance between groups with respect to first or second caesarean section. Regardless of whether the method of randomisation is by envelope or telephone there will be balance within centre.

## Interventions

There are five pairs of interventions being tested in this trial; however, each participating hospital will only take part in three of these five possible comparisons. The non-allocated comparisons and all other aspects of the caesarean section will be performed at the discretion of the surgeon. For example, for women randomised in a hospital which is taking part in the three comparisons: sharp vs blunt abdominal entry; double vs single layer uterine closure; and closure vs non-closure of the peritoneum; then it will be at the discretion of the surgeon as to whether there is exteriorisation of the uterus or not, and what suture material is used for uterine repair.

All allocated techniques should be performed unless there are overwhelming reasons not to do so. For example, at the time of surgery it may become apparent that there is no clear peritoneal layer overlying the uterus in a woman undergoing a second caesarean section. In these circumstances, it will not be possible to close this peritoneal layer during the repair. Other clinical circumstances may also change during the course of the surgical procedure after a particular intervention has been allocated. If the clinician performing the caesarean section is certain that a non-allocated intervention is necessary then this should be performed and the reasons for not complying with the allocated intervention recorded on the data collection form.

### 1. Blunt versus sharp abdominal entry

#### Sharp abdominal entry

The abdomen is entered using a scalpel to divide the abdominal skin. Each subsequent layer of the abdomen (fat, rectus sheath and parietal peritoneum) is then separately identified and divided using either a scalpel or scissors.

#### Blunt abdominal entry

The abdomen is entered using a scalpel to divide the abdominal skin. The scalpel is then used to divide the fat and rectus sheath in the midline and the rectus sheath incision is extended manually (without the use of a scalpel or scissors). The parietal peritoneum is then entered digitally and the defect enlarged manually.

For some women with a previous caesarean section, blunt abdominal entry may be impeded by scar tissue. If this is the case, surgeons should use whatever technique is necessary to effect abdominal entry with the minimum of risk to the well being of the woman. If during the blunt abdominal entry a scalpel or scissors are used to extend the original incision in the sheath or peritoneum, then this should be recorded as a sharp abdominal entry on the Data Collection Booklet.

### 2. Exteriorisation of uterus for repair versus intra-abdominal repair

#### Exteriorisation

Once the placenta has been delivered the uterus is drawn from the pelvis to rest on the anterior abdominal wall so that the uterine incision can clearly be visualised. The uterus is then repaired (with or without repair of the pelvic peritoneum) and the uterus is then returned to the pelvis.

If exteriorisation of the uterus is not possible because of pain, then intra-abdominal repair may be necessary. This should be recorded as intra-abdominal repair on the Data Collection Booklet and the reason for non-compliance with the allocated intervention given as "intra-operative pain".

#### Intra-abdominal repair

The uterine incision is repaired while in the pelvis.

### 3. Single versus double layer closure of the uterus

#### Double layer uterine closure

The uterine incision is closed with two layers of sutures. Each layer may be continuous, continuous locking, interrupted or any other accepted technique.

The first layer opposes the endometrial aspect of the uterine muscle layer and the second layer of sutures bring together the serosal layer ensuring haemostasis and complete apposition of the incision.

#### Single layer uterine closure

This technique involves bringing both edges of the uterine incision together with a single layer of sutures. This may be a continuous, continuous locking or an interrupted layer of sutures.

Haemostasis of the incision can be effected by using additional single or 'figure of eight' sutures as judged necessary by the surgeon regardless of the method of closure undertaken.

### 4. Closure or non-closure of the peritoneum

#### Closure of the peritoneum

Women allocated closure of the peritoneum should have the pelvic and parietal peritoneum closed. The techniques of closure, including the suture material used, will be at the discretion of the clinician.

In some women with a previous caesarean section, repair of the pelvic peritoneum, the parietal peritoneum or both may be impossible. If this is the case, surgeons should record this as non-closure of the peritoneum on the Data Collection Booklet including the reason for non-compliance.

#### Non-closure of the peritoneum

In women allocated non-closure, the peritoneum should not be closed.

For either closure or non-closure of the peritoneum, haemostasis should be affected as usual including, where necessary, the use of haemostatic sutures.

### 5. Chromic catgut versus Polyglactin-910 for uterine repair

#### Chromic catgut

The uterus will be repaired using No. 1 chromic catgut. This may be a continuous, continuous locking or an interrupted layer of sutures.

#### Polyglactin-910

The uterus will be repaired using No. 1 Polyglactin-910. This may be a continuous, continuous locking or an interrupted layer of sutures.

Haemostasis of the incision can be effected by using additional single or 'figure of eight' sutures as judged necessary by the surgeon.

Regardless of the type of suture material allocated, any unused sutures **should not **be used to effect repair of any other tissue layer. The standard materials used in each centre should be used to close the peritoneum (if performed), the sheath, the subcutaneous tissue (if closed) and the skin.

Use of the allocated suture material to close other tissue layers will render the randomised comparison of Polyglactin-910 versus chromic catgut invalid (particularly if there is differential use, i.e. the Polyglactin-910 is used and the chromic catgut is not used to close the sheath).

## Other aspects of clinical management

All other aspects of the operation, apart from those randomly allocated, will be determined by the attending surgeon and anaesthetist including the choice of suture materials and the techniques for repair used.

Women participating in the trial will be managed in whatever way seems best for them. Participation in the trial does not restrict the use of any other therapeutic or diagnostic procedures judged necessary by the attending clinician. This applies particularly to the use of analgesia, perioperative antibiotics or thromboprophylaxis. Current hospital practice should be continued, regardless of participation in the trial.

It is essential that the other aspects of the caesarean section operation remain constant regardless of those aspects which are allocated. So, for example, if it is the usual practice of the surgeon to infiltrate the skin edges with local anaesthetic for post-operative analgesia, this should continue for all women operated on by that surgeon regardless of whether the women is allocated sharp or blunt abdominal entry. The use of other interventions, such as per-rectum analgesia, should similarly be the same for women in each allocated group. Differential use of these co-interventions between randomised groups will render the comparisons of the trial invalid.

## Training in surgical techniques

Of the techniques being compared, training may be required in single or double layer uterine closure and in blunt and sharp abdominal entry. All the other techniques are familiar to all obstetricians in clinical practice in the participating centres.

Each participating centre will initiate, maintain and document a training programme which ensures that all personnel involved in the undertaking of caesarean sections are familiar with the techniques being compared before they enter women into the trial. Training will vary between countries according to the national standards of training in new surgical techniques employed by each participating country. For example, if the accepted standard for surgical training in a country is that operators must perform a certain number of procedures before they are judged to be competent in that procedure, then this process should be followed. If, however, the national standard is that operators are judged to be competent when a senior surgeon judges them to be competent, then this process should be followed. The accepted standard of surgical training in each centre will be determined at the start of the trial and specific documentation will be produced for each country to facilitate the recording of details of each operator 'accredited' to operate on women recruited to the trial. To facilitate training a film of all the interventions being tested in CORONIS will be provided to participating centres. In individual countries, visits by the Regional Co-ordinator to participating hospitals to teach specific surgical techniques may be required so that experience in the participating hospitals can be disseminated rapidly.

Each participating centre will appoint a senior obstetrician to ensure that only clinical staff competent in the various surgical techniques to be used in the trial are 'authorised' to operate. A list of these personnel will be kept by the local centre with a copy being kept by each Regional Trial Office.

## Trial eligibility

Women **ARE **eligible for trial entry if:

• They are undergoing delivery by lower segment Caesarean section through a transverse abdominal incision, *irrespective of fever in labour, gestational age or whether they have a multiple pregnancy*.

Women are **NOT **eligible if :

• There is a clear indication for a particular surgical technique or material to be used that prevents any of the allocated interventions being used,

*e.g. for a woman with a previous vertical abdominal incision it maybe considered inappropriate to do a transverse abdominal incision for this caesarean section. However, if a transverse incision is going to be performed the woman is eligible*.

• They have had more than one previous caesarean section.

• They have already been recruited into the trial during a previous pregnancy.

## Trial entry and consent

Each participating country and hospital, with advice from their research ethics committee, will decide how best to provide information to women about the trial and seek informed consent.

Information leaflets will be made available to local centres, in appropriate languages, which explain the justification for the trial, the process of trial entry and follow up. Once a woman becomes eligible, the trial should be discussed with her (and her partner as appropriate).

During this discussion the following points should be addressed:

• that consent is being sought for the woman's participation in a randomised controlled trial;

• the trial will compare different aspects of the caesarean section techniques, all of which are in common practice throughout the world;

• that the aspects being compared will be allocated at random;

• that participation in the trial is voluntary and declining to enter the trial will not affect the quality of the medical care the woman receives;

• that one hospital visit or home visit is required at six weeks after the birth of her baby;

• that she is free to withdraw her consent to participate in the trial at any time.

All women will be required to sign a formal consent form if they agree to participate in the trial. If women are not able to sign a consent form, the method of recording consent which is currently used in that setting will be used for the trial (for example, the recording of a thumb print from the woman on the consent form). If there is no objection to trial entry, a few brief details will then be recorded on the trial data collection form.

This information is collected for three reasons:

i. to check eligibility,

ii. to enable later (unbiased) description of the women studied,

iii. to assist follow-up.

## Measurement of outcomes

### Primary outcome

1. Death or maternal infectious morbidity (one or more of the following: antibiotic use for maternal febrile morbidity during postnatal hospital stay, antibiotic use for endometritis, wound infection or peritonitis) or further operative procedures; or blood transfusion.

*Note: although there are concerns about the use of composite outcomes, we believe that the advantage of increased precision of the estimate of short term morbidity out-weighs any potential disadvantages. All the outcomes included in this composite are associated with the primary objective of the trial and are likely to act in the same direction as each other thereby preventing any dilution of the treatment effect*.

### Secondary outcomes – all within 6 weeks of delivery unless stated otherwise

#### Clinical

1. Death.

2. Febrile morbidity.

*Note: any clinical diagnosis of fever >38°C made by a health professional and treated with antibiotics during postnatal hospital stay*.

3. Endometritis.

*Note: any clinical diagnosis of endometritis made by a health professional and treated with antibiotics during the first six weeks after the caesarean section. For events which occur after discharge from hospital this information will be gathered by interview with each woman six weeks after the caesarean section*.

4. Wound infection treated with antibiotics.

*Note: any antibiotics prescribed specifically for a wound infection. This information will be gathered by interview with each woman at six weeks after the caesarean section*.

5. Operative procedures on wound.

*Note: this will include any procedures on the wound because of infection, dehiscence or haematoma within the first six weeks following delivery*.

6. Pain.

*Note: this will be a subjective assessment of post-operative pain by the women at the time of hospital discharge and at six weeks. In addition a category of 'severe pain' will be recorded if the woman is prescribed additional analgesia over and above routine 24–48 hours after surgery*.

7. Blood transfusion.

8. Interventions used for severe primary post-partum haemorrhage (PPH).

*Note: this is defined as women who (a) require additonal uterotonics over and above routine, (b) where a brace suture is used (eg. B-Lynch suture), (c) where internal iliac artery, or other major artery, ligation is performed, (d) where balloon tamponade or uterine packing is performed or (e) where a hysterectomy is performed*.

9. Stillbirth after trial entry.

*Note: it is possible that a delay in abdominal entry associated with different methods may adversely affect neonatal outcome*.

10. Apgar score < 3 at five minutes.

11. Laceration of baby at time of caesarean section.

12. Death of the baby by six weeks of age.

13. Other severe maternal morbidity.

*Note: this will include events such as peritonitis, severe secondary post-partum haemorrhage, deep venous thromboses, pulmonary embolism, and sepsis within six weeks of delivery, etc*.

#### Health Service Utilisation

14. Duration of operation (from incision to closure).

15. Duration of hospital stay post-caesarean section.

16. Duration of stay in Intensive Care Unit post-caesarean section.

17. Number and duration of re-admissions to hospital within 6 weeks of the caesarean section.

## Data collection

Information will be collected at the following times:

• at trial entry;

• immediately following the operation;

• at discharge from hospital (including transfer to another hospital);

• at six weeks after delivery.

Information at trial entry including eligibility and maternal characteristics will be collected from the hospital notes onto the trial data collection booklet. Once the caesarean section is complete information about the procedure will be recorded by the surgeon who performed the operation. At discharge from hospital (or transfer to another hospital) staff on the postnatal ward will record information about the trial outcomes from hospital records.

In addition, all women will be seen at six weeks postpartum to determine whether they have experienced any of the other trial outcomes. Follow-up will be achieved in a variety of ways: women will be encouraged to return to the hospital at six weeks postpartum for a routine check-up at which point trial specific information can also be requested. Women who do not return at six weeks will be sought and interviewed by appropriately trained personnel employed by the trial either by telephone (if feasible) or by home visits.

To facilitate the accurate recording of clinical diagnoses and treatments after hospital discharge women will be provided with a medical card at discharge from hospital. Women will ask health professionals who they see after hospital discharge to record any diagnoses made and record the treatment prescribed. Although this is unlikely to be used on all occasions, it should enhance the quality of the information obtained at the six week interview.

Sufficient descriptive information will be collected about the participating women in the trial to allow further follow-up if funds can be secured in the future. Of particular interest in the future is the effect that operative techniques may have on future pregnancies, specifically problems with conception, antenatal complications such as the incidence of abruption, mode of delivery especially 'in-labour' caesarean sections, placental problems, subsequent wound complications and stillbirths.

## Analysis

The analysis of this trial will be by 'intention-to-treat'. Therefore women will be analysed by the groups into which they were randomised regardless of what interventions they received.

For the analysis, the risk ratio for each outcome will be estimated for each of the five surgical techniques. A 95% confidence interval will be used for the main outcome although any results which are of "borderline" statistical significance will be treated cautiously owing to the five comparisons being made. For secondary outcomes, 99% confidence intervals will be calculated in order to take account of the number of secondary outcomes.

The focus of the analysis will be on the main effects of the five interventions. However, pairwise interactions between any two interventions will be tested for and the results will be stratified accordingly if interactions are found. Alternatively, if risk differences provide a better fit to the data than risk ratios, then these will be presented instead. It is recognised that the study has low power to detect interactions, therefore, an interaction test which is not statistically significant does not mean that there really is no interaction. Alternatively, there are 24 possible interaction pairs which can be tested for, so "significant interactions" may arise by chance. Hence, the interaction results will be interpreted cautiously together with other evidence such as biological plausibility and consistency (e.g. across countries).

In addition, pre-defined subgroup analysis will be performed based on outcomes stratified by:

i. 'In-labour' or not 'in-labour' caesarean section,

ii. Single and multiple pregnancy,

iii. HIV positivity (positive, negative, not known),

iv. Number of previous caesarean sections (none or one),

v. Presence of intrapartum fever,

vi. Type of anaesthesia (local, regional, general),

vii. Experience of the operator (number of years experience in obstetrics),

viii. Centre or Country (numbers permitting).

Further exploratory analysis will be performed to generate hypotheses for future testing. This will include the effects of other interventions and techniques used during the operation and the effect these have on the outcomes.

### Interim analyses: the Data Monitoring Committee

For the trial a Data Monitoring Committee (DMC) has been established. This is independent of the trial organisers. The DMC will meet before the trial commences to agree their terms of reference and the method of working using the DMC Charter [26]. During the period of recruitment to the trial, interim analyses will be supplied, in strict confidence, to the DMC, together with any other analyses the DMC may request. The data will be supplied to the Chair of the DMC as frequently as he requests. Meetings of the committee will be arranged periodically, as considered appropriate by the Chair but these meetings will occur at least once a year after trial recruitment commences.

In the light of interim data, and other evidence from relevant studies (including updated overviews of the relevant randomised controlled trials), the DMC will inform the Trial Steering Committee, if in their view there is proof beyond reasonable doubt that the data indicate that any part of the protocol under investigation is either clearly indicated or contra-indicated, either for all women or for a particular subgroup of trial participants. A decision to inform the Trial Steering Committee of such a finding will in part be based on statistical considerations.

Appropriate criteria for proof beyond reasonable doubt cannot be specified precisely. A difference of at least 3 standard errors in the interim analysis of a major endpoint may be needed to justify halting, or modifying, such a study prematurely. If this criterion were to be adopted, it would have the practical advantage that the exact number of interim analyses would be of little importance, and so no fixed schedule is proposed [27]. Unless modification or cessation of the protocol is recommended by the DMC, the Trial Steering Committee, collaborators and administrative staff (except those who supply the confidential information) will remain ignorant of the results of the interim analysis. Collaborators and all others associated with the study may write through the International Co-ordinating Centre to the DMC, to draw attention to any concern they may have about the possibility of harm arising from the treatment under study, or about any other matters that may be relevant.

### Data Monitoring Committee membership

Professor Zarko Alfirevic.

Dr Susan Bewley.

Dr Oona Campbell.

Professor Jon Deeks.

Professor Florence Mirembe.

## Serious Adverse Event reporting

Serious Adverse Events should be reported to the CORONIS Regional Trial Office within 48 hours. The Regional Trial Office will then notify the International Co-ordinating Centre in Oxford who will then report it to the Chair of the DMC and the relevant research ethics committees, with a summary of the previously reported events, within 15 days. As all of the surgical techniques being tested in this trial are used throughout the world there are no Serious Adverse Events which would be anticipated as a unique consequence of participation in the trial. We would, however, expect the following to be reported:

• All maternal deaths;

• Severe haemorrhage (requiring transfusion of six or more units of blood);

• Repeat laparotomy or hysterectomy;

• Admission to Intensive Care Unit;

• Any other serious unexpected adverse events.

## Sample size

In our pilot survey of 855 consecutive women undergoing caesarean section from 9 hospitals, the incidence of serious maternal morbidity before discharge from hospital was 11.6% (95% CI: 9.6–13.9). Inclusion of outcomes up to six weeks after the caesarean section is therefore likely to increase the incidence of the primary outcome to at least 15%. Assuming the incidence of the primary outcome is 15% in one group, then 7,586 women would need to be randomised to give 80% power to detect a risk ratio between the two groups of 0.85 (i.e. a reduction in incidence to 12.75%; (Table 7). A trial of this size will have in excess of 95% power to detect a risk ratio of 0.80 (e.g. to compare 15% with 12%).

**Table 7:** Sample size

**Incidence in control group**	**Incidence in intervention group**	**Risk ratio**	**Total sample size needed for analysis of each comparison**
15%	18%	1.20	4,936
15%	17%	1.13	10,746
15%	13%	0.87	9,646
**15%**	**12.75%**	**0.85**	**7,586**
15%	12%	0.80	4,204
15%	11%	0.73	2,316

Sample sizes required for 15% incidence of primary outcome in control group, with 80% power

In order to ensure that there are 7,586 women included in the analysis of each comparison, a larger number of women need to be recruited. First, in order to carry out five comparisons when each centre takes part in only three, 12,668 women (7,586 × 1.67) would need to be recruited. Second, allowing for 15% loss to follow-up, about 14,904 women (12,668/0.85) would need to be recruited. Hence, the final target sample size is 15,000 women (Table 7).

## Feasibility

Women will be recruited by local clinicians prior to their caesarean section. Each country will have a Regional Co-ordinator to assist individual centres with data collection. In the pilot study, data were collected on 855 eligible women in 9 centres over 40 days (7,800 women/year). Assuming that 50% of eligible women will consent to join the trial, between 12 and 16 centres should be sufficient to complete the trial in three years. The sizes of centres will vary in different countries, however, and therefore more centres may be needed. The Regional Co-ordinators of each country will recruit sufficient hospitals to complete recruitment within the trial's timescale.

In order to ensure that a similar number of women are included in each of the five comparisons, the centres will need to be balanced according to the "combinations" of the three interventions. For example, if the small centres were recruiting to intervention 1 (i.e. they were allocated the following combinations of interventions: 123; 124; 125; 134; 135; 145) and the large centres were recruiting to intervention 5 then the study may not have enough power to detect an effect for intervention 1. Therefore, the combinations of the three interventions will be allocated so that each intervention is randomised to a similar number of women.

## Publication, presentation and dissemination of results

The Chief Investigator will co-ordinate dissemination of data from this trial.  To safeguard the scientific integrity of the trial, all proposals for subsidiary studies linked to the CORONIS Trial should be presented to the Project Management Group for approval.  All publications from subsidiary studies using data from the original analyses must be submitted to the Trial Steering Committee (TSC) for review before publication. Data from the trial will not be presented in public before the main results are published without the prior consent of the TSC (see Organisation below).     

The success of the trial depends on a large number of clinicians and participants. For this reason chief credit for the results will not be given to the committees or central organisers but to all who have collaborated and participated in the study.    

Authorship at the head of the primary results paper will take the form ‘The CORONIS Trial Collaborative Group’. This avoids giving undue prominence to any individual. All contributors to the study will be listed at the end of the report, with their contribution to the study identified.  This will include all members of the Trial Steering Committee, the Project Management Group, the International Co-ordinating Centre, the Regional Trial Offices and local investigators at all participating sites.    

Those responsible for other publications reporting specific aspects of the trial may wish to utilise a different authorship model, such as “[name], [name] and [name] on behalf of The CORONIS Trial Collaborative Group”.    

Decisions about authorship of additional papers will be discussed and agreed by the trial investigators. Authorship of these papers should follow standard academic rules and should be discussed and agreed by the trial investigators.  A description of the uniform requirements for manuscripts submitted to biomedical journals is available online (October 2004; ).  

The criteria for authorship are:  

· Substantial contributions to conception and design, or acquisition of data, or analysis and interpretation of data  

· Drafting the article or revising it critically for important intellectual content  

· Final approval of the version to be published.   All authors should fulfil all of the criteria for authorship.   

Acquisition of funding, collection of data or general supervision alone does not of itself justify authorship.  

Presentations given about CORONIS should include an acknowledgement of the contribution of all the trial investigators and their organisations and of other collaborators and participants.  

## Organisation and governance

The protocol received ethics approval in the UK from OXTREC: Oxford Tropical Research Ethics Committee. Date of approval: 5th March 2007. OXTREC Reference number: 013-06. Each country will obtain ethics approval prior to recruitment commencing.

The trial will be run according to the MRC Good Clinical Practice guidelines and local data protection requirements. The trial will be monitored on a day-to-day basis in the International Co-ordinating Centre at the National Perinatal Epidemiology Unit (NPEU). This group reports to the Trial Steering Committee which is responsible to the UK Medical Research Council and the University of Oxford.

The Trial Steering Committee will oversee the conduct of the trial and will work to a charter agreed by all members.

### Trial Steering Committee

The specific tasks of the TSC are:

1. to approve the study protocol,

2. to approve necessary changes in the protocol based on considerations of feasibility and practicability,

3. to receive the report from the Data Monitoring Committee,

4. to resolve problems brought to it by the International Co-ordinating Centre,

5. to review and approve add-on studies, including higher degrees, study reports and papers for publication.

Each Regional Trial Office will report to a Regional Co-ordinator, who in turn will report to the Project Management Group via the project funded staff based at the NPEU.

Each centre will have a participating clinician, and further staffing will vary depending on the amount of community follow-up required.

### Trial Steering Committee membership

See Table 8.

**Table 8:** 

Felicity Ashworth	Obstetrician	Independent member
Peter Brocklehurst	Perinatal Epidemiologist	Chief Investigator
Simon Cousens	Statistician	Independent member
Debbie Chippington-Derrick	Consumer Representative	Independent member
Barbara Farrell	Trial Director	Observer
James Neilson	Obstetrician	Chair – Independent member
Manorama Purwar	Obstetrician	Independent member
Ed Juszczak	Trial Statistician	Observer
Fiona Russell	Programme Manager	MRC representative
Catriona Waddington	Consumer Representative	Independent member
Investigator representative	Obstetrician	Investigator – in rotation

### International Co-ordinating Centre

The National Perinatal Epidemiology Unit at the University of Oxford will be the International Co-ordinating Centre and will be responsible for the day to day running of the trial. The functions include:

i. recruitment of participating centres via the Regional Co-ordinators,

ii. distribution and supply of data collection forms and other appropriate documentation for the trial to the Regional Trial Offices,

iii. central data management,

iv. data cleaning,

v. data analysis,

vi. collection and dissemination of adverse event data,

vii. organising and servicing the Data Monitoring Committee and the Trial Steering Committee.

## Regional Trial Offices

Each country will have a trial office with responsibility to the Regional Co-ordinator. There will be two trial offices in India, one in Delhi and one in Vellore. The trial office will be responsible for one or more participating hospitals in their country or region. The responsibilities of each trial office include:

i. recruitment of participating centres,

ii. distribution and supply of data collection forms and other appropriate documentation for the trial,

iii. local data management,

iv. data entry and cleaning,

v. collection of adverse event data and communication of this to the International Co-ordinating Centre and elsewhere as required,

vi. coordinate documentation of training undertaken by operators in each of the participating centres.

## Participating sites

Local co-ordination in participating sites is the responsibility of a lead clinician in the participating hospital who is responsible for ensuring the smooth running of the trial in their unit.

The specific responsibility of the local co-ordinators will be to:

i. be familiar with the trial,

ii. liaise with their Regional Trial Office,

iii. ensure that all medical and midwifery staff involved in the care of pregnant women are well informed about the trial,

iv. ensure that mechanisms for recruitment of eligible women (including information material) are in place, monitor their effectiveness, and discuss reasons for the non-recruitment of any eligible women with relevant staff,

v. ensure that supplies of data collection booklets are always available, that they are completed and returned to the Regional Trial Office promptly, and to deal with any queries arising,

vi. notify the Regional Trial Office of any serious adverse events,

vii. facilitate other aspects of local collaboration as appropriate,

viii. make all data available for verification, audit and inspection purposes as necessary,

Argentina

Hospital Nacional Profesor Alejandro Posadas, Illia y Marconi, Haedo, Buenos Aires, Argentina.

Hospital Interzonal General de Agudoa Dr. Jose Penna, Lainez 2400, Buenos Aires, Argentina.

Hospital Dr. José María Cullen, Sante Fe, Argentina.

Hospital J. B. Iturraspe, Sante Fe, Argentina.

Hospital Regional Dr. Ramón Carrillo, Belgrano 2273, Santiago del Estero, Argentina.

Ghana

Komfo Anokye Teaching Hospital, Kumasi, Ghana.

India – (north) Delhi

All India Institute of Medical Sciences (AIIMS), Ansari Nagar, New Delhi, India.

Maulana Azad Medical College & Lok Nayak Hospital, New Delhi, India.

Lady Hardinge Medical College & Sucheta Kriplani Hospital, New Delhi, India.

University College of Medical Sciences & GTB Hospital, Shahdara, New Delhi, India.

India – (south) Vellore

Christian Medical College Hospital, Vellore, India.

Kenya

Kenyatta National Hospital, Nairobi, Kenya.

Pakistan

Fatima Bai Hospital, Business Recorder Road, Karachi, Pakistan

Liaquat National Hospital, Stadium Road, Karachi, Pakistan

Sobhraj Maternity Hospital, City District Government, Karachi, Pakistan

Countess of Dufferin Hospital, Chand Bibi Road, Kharadar, Karachi, Pakistan

Sudan

Soba University Hospital, Alamarat, Khartoum, Sudan

Omdurman Maternity Hospital, Omdurman, Sudan

## Abbreviations

DMC Data Monitoring Committee

ICC International Co-ordinating Centre

NPEU National Perinatal Epidemiology Unit

PPH post partum haemorrhage

RR risk ratio

RTO Regional Trial Office

SD standard deviation

TSC Trial Steering Committee

VAS visual analogue scale

WMD weighted mean difference

## Competing interests

The author(s) declare that they have no competing interests.

## Authors' contributions

Members of The CORONIS Trial Collaborative Group were involved in the conception and design of the study and are Regional Co-ordinators overseeing participating sites in their country.

Professor Peter Brocklehurst, National Perinatal Epidemiology Unit, Oxford, drafted the manuscript.

All members of the CORONIS Collaborative Group edited the manuscript and read and approved the final manuscript.

Members of The CORONIS Trial Collaborative Group are:

Dr Edgardo Abalos,

Centro Rosarino de Estudios Perinatales, Rosario, Argentina

Dr Victor Addo,

Komfo Anokye Teaching Hospital, Kumasi, Ghana

Dr JB Sharma,

All India Institute of Medical Sciences, Delhi, India

Dr Jiji Matthews

Christian Medical College and Hospital, Vellore, India

Professor James Oyieke

Kenyatta National Hospital, Nairobi, Kenya

Dr Shabeen Naz Masood

Fatima Bai Hospital, Karachi, Pakistan

Professor Mohamed A El Sheikh

University of Khartoum, Khartoum, Sudan

## Pre-publication history

The pre-publication history for this paper can be accessed here:


